# Recombination-mediated escape from primary CD8+ T cells in acute HIV-1 infection

**DOI:** 10.1186/s12977-014-0069-9

**Published:** 2014-09-12

**Authors:** Adam John Ritchie, Fangping Cai, Nicola MG Smith, Sheri Chen, Hongshuo Song, Simon Brackenridge, Salim S Abdool Karim, Bette T Korber, Andrew J McMichael, Feng Gao, Nilu Goonetilleke

**Affiliations:** Blavatnik School of Government, University of Oxford, Oxford, OX1 4JJ UK; Department of Medicine, Duke University, Durham, NC 27710 USA; Weatherall Institute of Molecular Medicine, University of Oxford, Oxford, OX3 9DS UK; University of KwaZulu-Natal, Durban, 4041 South Africa; Theoretical Division, Los Alamos National Laboratory, Los Alamos, NM 87545 USA; The Sante Fe Institute, Sante Fe, NM 87501 USA; National Engineering Laboratory for AIDS Vaccine, College of Life Science, Jilin University, Changchun, Jilin, China; Department of Microbiology & Immunology, University of North Carolina at Chapel Hill, Chapel Hill, NC 27599 USA

**Keywords:** HIV-1, T cell, Multiple infection, Recombination, Immunodominance, Acute infection

## Abstract

**Background:**

A major immune evasion mechanism of HIV-1 is the accumulation of non-synonymous mutations in and around T cell epitopes, resulting in loss of T cell recognition and virus escape.

**Results:**

Here we analyze primary CD8+ T cell responses and virus escape in a HLA B*81 expressing subject who was infected with two T/F viruses from a single donor. In addition to classic escape through non-synonymous mutation/s, we also observed rapid selection of multiple recombinant viruses that conferred escape from T cells specific for two epitopes in Nef.

**Conclusions:**

Our study shows that recombination between multiple T/F viruses provide greater options for acute escape from CD8+ T cell responses than seen in cases of single T/F virus infection. This process may contribute to the rapid disease progression in patients infected by multiple T/F viruses.

**Electronic supplementary material:**

The online version of this article (doi:10.1186/s12977-014-0069-9) contains supplementary material, which is available to authorized users.

## Background

HIV-1 specific CD8+ T cells are first detected prior to peak viremia and expand concomitantly with decline of acute plasma virus load (pVL) [1,2]. HIV-1 peptide epitopes are presented to CD8+ T cells in complex with polymorphic human leukocyte antigen (HLA)-I molecules. Certain HLA allotypes are associated with lower pVL setpoints and better clinical outcomes, suggesting an important role for CD8+ T cells in control of HIV-1 replication [3-6]. HIV-1 specific CD8+ T cells are also a major selective force in viral evolution *in vivo* [7,8] and can select non-synonymous virus escape mutants in and around the reactive epitope, that wholly or partially ablate T cell reactivity, within weeks of infection [9,10]. The timing of escape for each epitope is not random and is heavily impacted by the relative immunodominance of an individual CD8+ T cell response and the Shannon entropy, or population variability, of the targeted epitope [10,11].

HIV-1 infection with a single transmitted/founder (T/F) virus occurs in around 80% of heterosexual infections [12-14]. The proportion of multiple T/F viruses initiating infection increases in other groups, such as men who have sex with men and intravenous drug users. Infection with multiple T/F viruses is linked to factors that are known to increase overall transmission rates, such as higher risk sex acts and other concurrent sexually transmitted infections [12,15-19]. Several studies have associated infection with multiple HIV-1 T/F viruses, multiple subtypes, and/or a diverse virus population, with higher pVL setpoint, faster CD4+ T cell decline, earlier need for anti-retroviral therapy and a worse prognosis for the infected individual [14,20-24].

The emergence of recombinant viruses results from infection of a cell with two or more different viruses [25]. HIV-1 is highly recombinogenic [26] and HIV-1 recombination has been observed in patients infected with multiple viruses within weeks-months of infection [12,14,15,17]. Although none of these acute-phase studies have experimentally linked the emergence of recombinants to immune responses, several mathematical models have suggested that recombination may impact escape from CD8+ T cell responses [27,28]. Such associations have been suggested in one study of superinfection during the chronic stage of HIV-1 infection [29].

Here we report on a subject infected with two T/F viruses. We find that differential T cell targeting of the two T/F viruses drives accelerated recombination-mediated escape in acute infection.

## Results

### Acute HIV-1 replication in subject CH078

**Figure 1:**
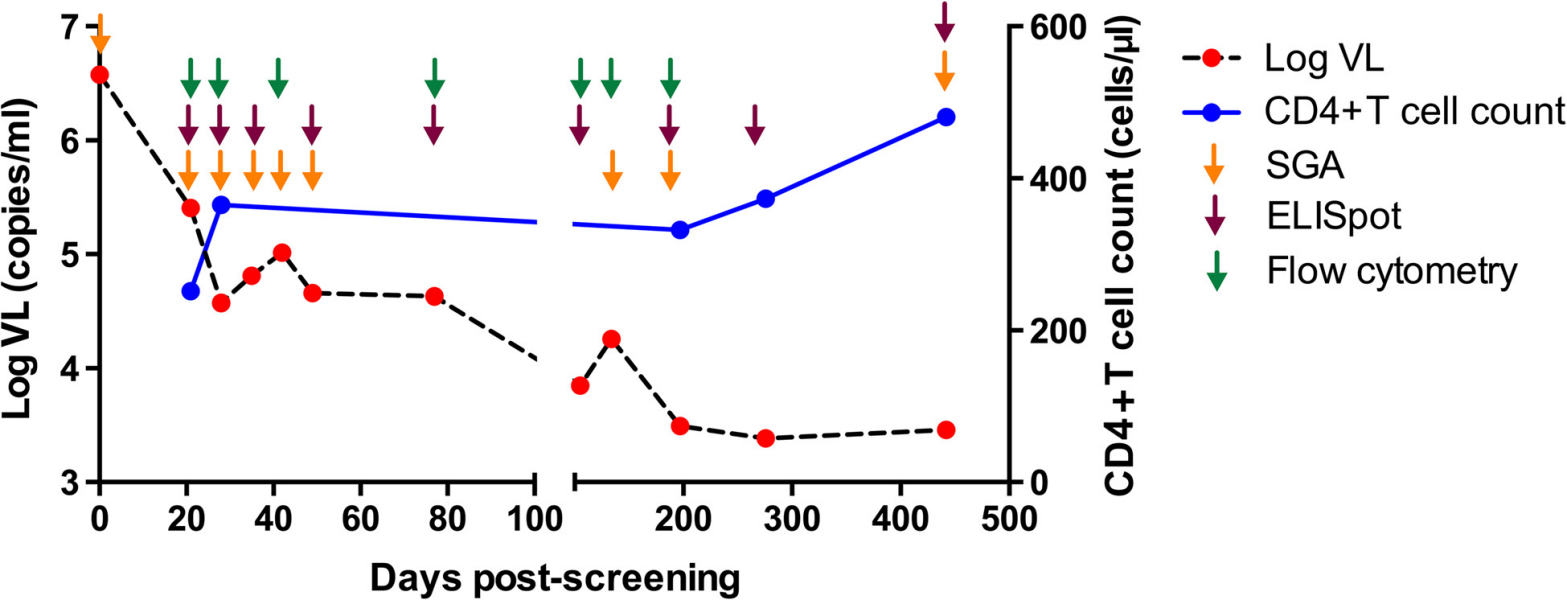
**Clinical data and experimental protocol for patient CH078.** CH078 was HIV-1 viral RNA positive, antibody negative (Fiebig I/II) at screening. The plasma VL (red points and black line) and CD4+ T cell counts (blue points and line) are shown. pVL declined rapidly by ~2 log within the first 28 days from screening. Next pVL stabilized from days 28–77, followed by another period of slower pVL decline of ~1 log over several months to establish a setpoint of 3,520 copies/ml around 6 months post-screening. Samples were used for SGA sequencing (orange arrows), T cell ELISpot (purple arrows), and flow cytometry (green arrows) at the timepoints indicated.

Subject CH078 was detected in acute HIV-1 infection stage Fiebig I/II (seronegative, pVL= 3 748 087 copies/ml), near peak viremia [30,31]. Genital ulcer disease, which has been associated with higher risk of HIV-1 transmission [32], was diagnosed at enrolment, 3 weeks later. From peak viremia, his pVL declined rapidly by ~2 log within the first 28 days from Fiebig I-II, then stabilized, even increasing slightly over the next 7 weeks (days 28–77). This was followed by a period of slower pVL decline of ~1 log over several months to establish a setpoint of 3,520 copies/ml around 6 months post-screening (Figure 1). CD4+ cell counts increased from a nadir of 251 cells/μl, 21 days post-screening and remained >300 cells/μl over the rest of the study period (441 days total) (Figure 1). His HLA type (A*01, A*30, B*42, B*81, Cw*17, Cw*18) included the protective HLA B*81 allele. In accordance with local clinical practice guidelines applicable at the time, he was not initiated on antiretroviral therapy during the course of this study.

### Patient CH078 was infected with two T/F viruses

Single genome amplification (SGA) and sequencing of overlapping 5′ and 3′ halves of HIV-1 genomes from subject plasma were performed at nine time points from screening to 441 days post-screening (Figure 1). This approach [13], allowed for analysis of recombination events. Fifty, 3′-half genome sequences were analyzed at screening (Fiebig I-II) giving > 90% confidence to detect virus variants at the 5% level [12]. Analysis identified (Additional file 1: Figure S1), a ‘major’ (96%) predominating virus with the other T/F ‘minor’ accounting for the remainder of the viral populations. These viruses were highly related (1.2% nucleotide differences in *env*) suggesting transmission from a single donor. The greatest variability was observed in Nef which differed by 10.7% at the amino acid (aa) level (Table 1). Two viral lineages were also detected in the 5′ side of the virus; however the presumed minor population (2 of 11 sequences) was not detected until 28 days post-screening (Additional file 1: Figure S1). Therefore, the 5′ half of the genome sequence for the minor T/F virus could not be reliably inferred and differences between the major and minor T/F viruses in the 3′ half genome were used to track changes in relative viral frequencies and recombination.

**Table 1:** **The sequence differences between the two T/F viruses in CH078 for each gene in the 3′ half viral genome**

**Gene**	**Nucleic acid (%)**	**Amino acid (%)**
Vif	0.7	1.0
Vpr	0.3	0.0
Tat	0.0	0.0
Rev	0.6	0.9
Vpu	0.8	0.0
Env	1.2	2.1
Nef	6.4	10.7

The first recombinant viruses were detected at day 35 from Fiebig I/II, as pVL initially stabilized (day 28–77). Recombinants then rapidly increased in frequency, reaching 44% of all sequences at day 42 and representing 100% of all sequences at day 77 and all subsequent timepoints (Additional file 1: Figure S1).

### Primary HIV-1 specific CD8+ T cell responses target both viruses

We examined whether acute T cell responses were associated with virus escape by mutation and recombination. HIV-1 specific T cell responses were comprehensively mapped in IFN-γ ELISpot assays using overlapping peptides that matched and spanned both the major and minor T/F viruses, and optimal epitopes subsequently defined experimentally. All T cell responses were CD8+ restricted and dominated by a central memory CD45RO + CD27+ phenotype (Additional file 2: Figure S2).

**Figure 2:**
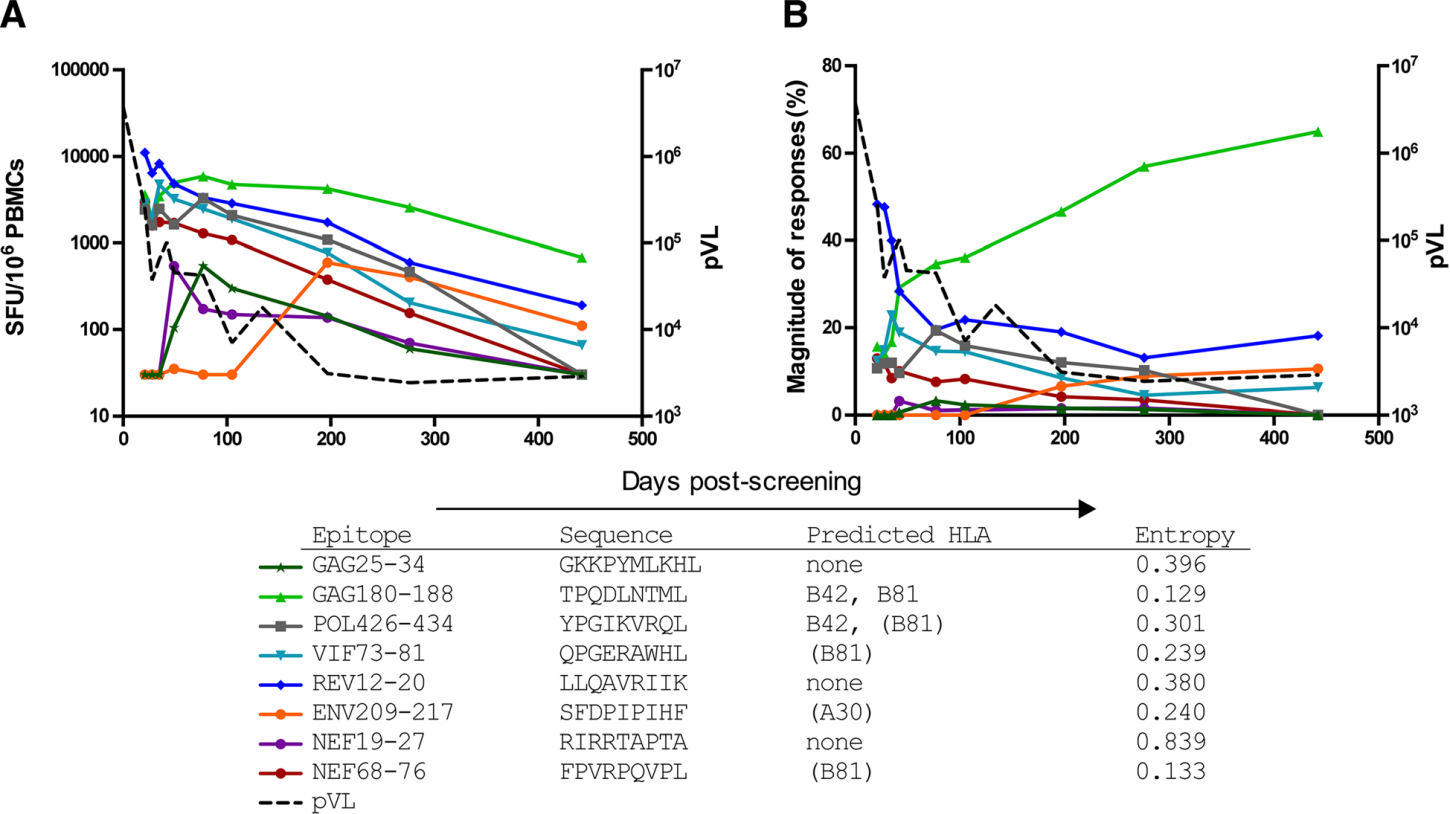
**High frequency HIV-1 specific T cell responses were induced in acute infection in CH078.** *Ex vivo* IFN-γ ELISpots were performed on PBMCs from CH078 between 21 and 442 days post-screening (Fiebig I/II). Solid colored lines represent individual T cell responses and are plotted as absolute magnitude **(A)** or % of total magnitude **(B)**. The plasma virus load (pVL) of CH078 is plotted as a dotted line on each graph. A T cell response was considered positive if it was ≥30 SFU/10^6^ cells and ≥4× background. All data represent the background subtracted mean spot forming units (SFU) per million PBMCs. For each response the epitope name based on HXB2 aligned amino acid position within individual proteins, the experimentally confirmed sequence, HLA restriction and epitope entropy are listed below the figure. Parentheses around HLA types indicate the HLA type predicted for the epitope based on binding motif as opposed to experimentally confirmed HLA restriction.

**Figure 3:**
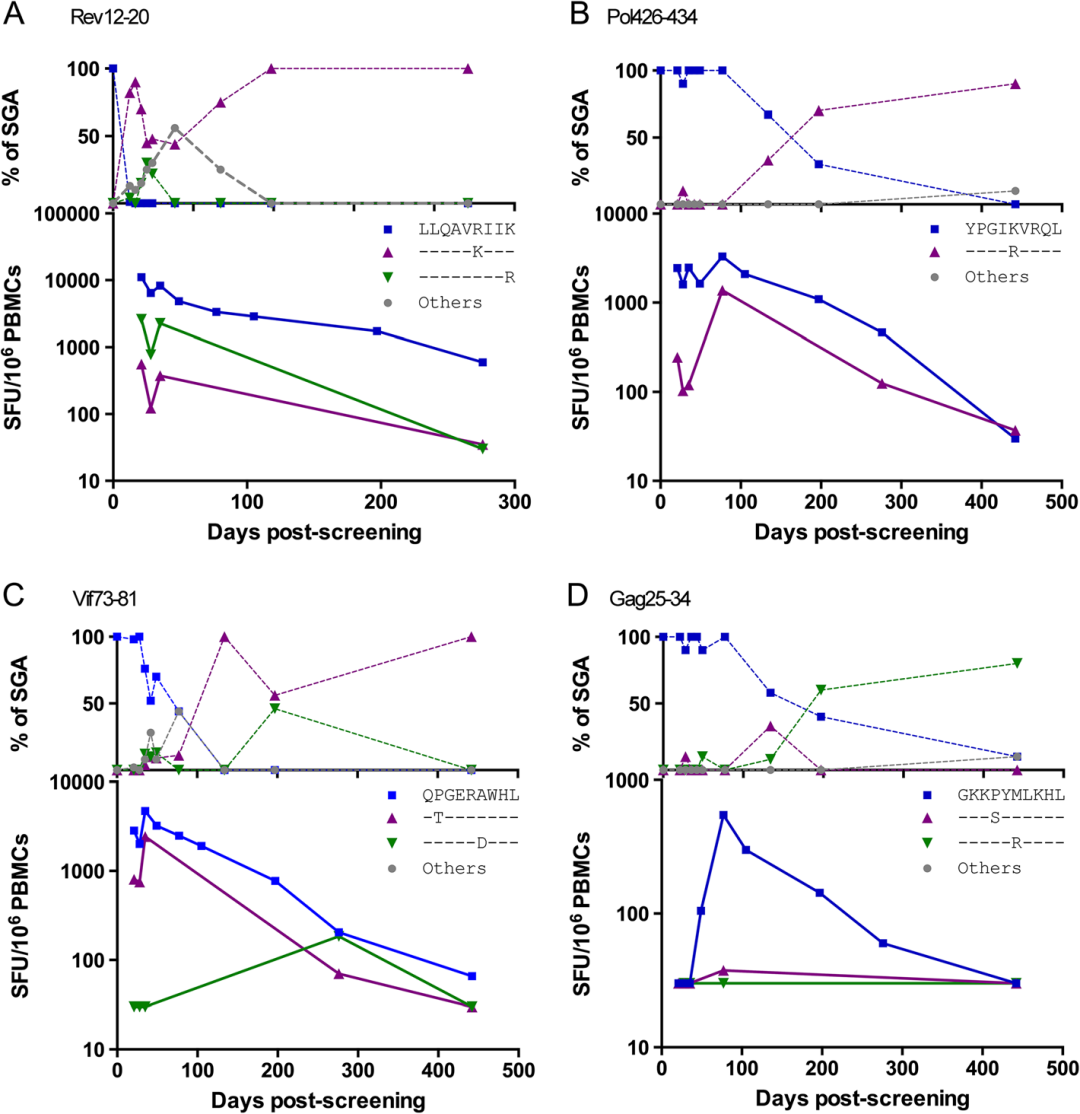
**CD8+ T cells targeting shared epitopes escaped via accumulation of variants in the reactive epitope.** The epitopes Rev12-20 **(A)**, Pol426-434 **(B)**, Vif73-81 **(C)**, and Gag25-34 **(D)** were shared between the major and minor T/F viruses and showed escape. The top graph in each figure represents the changing frequencies of each epitope as measured by SGA sequencing over time. The bottom graph in each figure represents the absolute magnitude of the T cell responses targeting all tested variants of the epitope, measured using *ex vivo* IFN-γ ELISpots. Each figure has a different scale on the Y axis for the bottom graph. Data for the T/F epitope are shown using squares, data relating to the escape variants tested in T cell assays are shown as triangles, while in the top graph only, other epitope variants (neither T/F nor tested escape variants) are shown as gray circles (others). Screening corresponds to Fiebig I/II.

Five primary HIV-1 specific T cell responses were detected when pVL was rapidly declining in acute infection (Figures 1 and 2A) of which four (Gag180-188, Pol426-434, Vif73-81, Rev12-20) targeted identical epitopes in the major and minor viruses (shared epitopes). Variable escape kinetics were observed following the accumulation of non-synonymous mutations within the targeted epitopes (Figure 3). The fifth T cell response targeted a 9-mer epitope (Nef68-76) that varied between the two T/F viruses by 1 aa at position 71 (variable epitope).

As infection progressed, three new HIV-1 specific T cell responses emerged, two targeting shared epitopes (Gag25-34, Env209-217) (Figure 2). The third targeted a variable 9-mer epitope (Nef19-27), which differed between the two T/F viruses at 2 aa residues at positions 25 and 26.

### Escape from T cell responses by virus recombination

**Figure 4:**
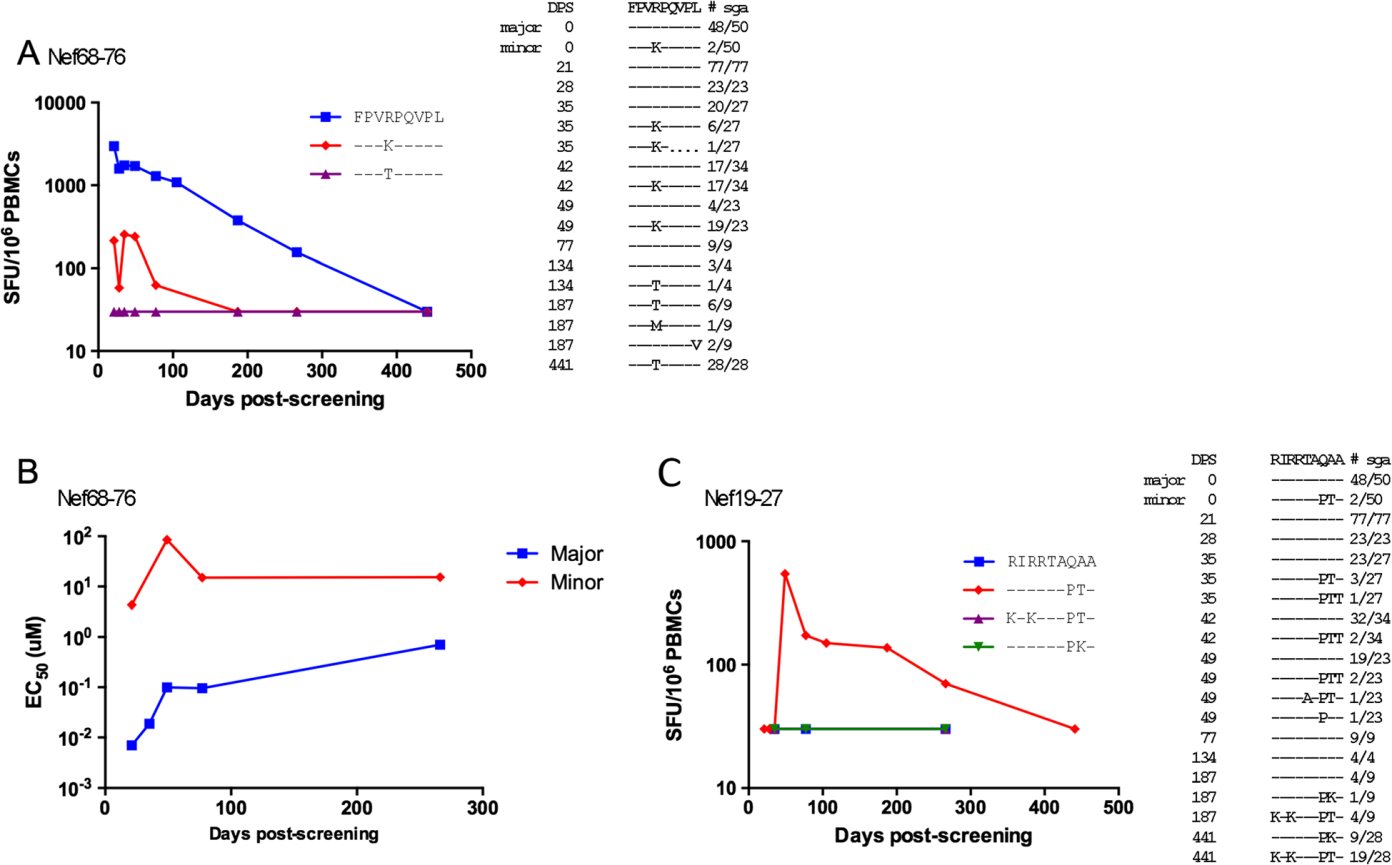
**CD8+ T cell responses differentially recognized Nef epitopes in the 2 T/F viruses.** The epitopes Nef68-76 **(A)** and Nef19-27 **(C)** varied between the major and minor T/F viruses. T cell responses were measured by *ex vivo* IFN-γ ELISpots with responses against the major T/F shown by blue squares and responses against the minor T/F shown as red diamonds. T cell responses to subsequently emerging escape variants are shown as triangles. To the right of these graphs, the changes in each epitope are shown over time. The EC_50_ of the Nef68-76 T cell response targeting the major variant was lower than that of the response targeting the minor variant **(B)**. dps = days post-screening (Fiebig I/II).

The CD8^+^ T cell response that targeted the variable Nef68-76 epitope recognized the major T/F epitope with greater magnitude (>10-fold), higher functional avidity (Figure 4A and B), and an increased proportion of reactive cells producing 3 or more cytokines when compared with the minor virus epitope (Additional file 3: Figure S3).

Within 50aa upstream of the Nef68-76 reactive epitope, a second Nef specific T cell response, Nef19-27, also recognized an epitope that was variable. In contrast to the Nef68-76 T cell response, the Nef19-27 response detected the minor virus and not the corresponding major virus epitope (Figure 4C). The minor virus existed at only 4% at Fiebig I-II and therefore the level of presentation of this epitope would have been very low relative to the corresponding major epitope. By day 35, the minor Nef19-27 epitope was found in 15% of sequences but no T cell response was detectable in ex vivo assays. However, at the next timepoint (day 49) when cells were available, this T cell response was detected strongly at > 500 SFU/10^6^ cells, suggesting this T cell response emerged between day 35 and 49.

**Figure 5:**
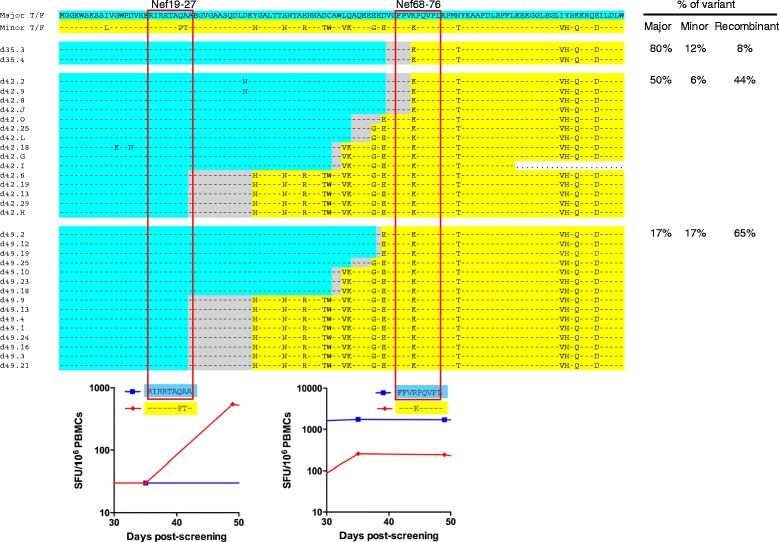
**HIV-1 recombination can afford rapid escape from acute CD8+ T cell responses.** The first 114 amino acid region of Nef spanning the two variable T cell epitopes in CH078 were analyzed. The recombination breakpoints between the epitopes are shown. The major and minor T/F consensus sequences are displayed above the individual sequences. Sequences derived from the major T/F are highlighted in blue and sequences from the minor T/F are highlighted in yellow, while the regions in which recombination probably occurred are indicated in grey. The patient ID and days post screening (dps) which corresponded to Fiebig I/II are followed by individual sequence identifiers. The 2 epitopes are outlined by red boxes. Graphical inserts beneath each epitope indicate the magnitude of T cell responses targeting the major and minor T/F variant of each epitope (data derived from Figures 4A and C). On the right, the percentage of the recombinant viruses at each timepoint is shown. Non-recombinant viruses are broken down in the % major or minor virus. Note, at day 35, two 3′ sequences (of 27) with large deletions were excluded from analysis.

Given that these Nef-reactive T cell responses each targeted the major and minor T/F viruses in a reciprocal manner, we explored whether HIV-1 could evade these two T cell responses through recombination. Amino acid variation between the two T/F viruses was also found in the region (114aa) surrounding these epitopes (Figure 5), which allowed us to unequivocally distinguish the recombinants from the T/F viruses and identify recombination breakpoints. No recombination was detected before day 35 (Additional file 4: Figure S4). At day 35, 2 out of 25 (8%) of sequences were recombinants, and both of these shared identical breakpoints (Figure 5, Additional file 4: Figure S4). Recombinants increased to 44% and 65% at days 42 and 49 respectively, with different breakpoints identified in different sequences (Additional file 4: Figure S4). From days 35 to 49, a total of 32 sequences had a recombination breakpoint between the two epitopes, and in all cases were found to be carrying the epitopes least targeted by T cell responses (Figure 5). Five distinct breakpoints were identified in this population of 32 sequences (Figure 5). This suggests that these viruses became dually resistant to the Nef-reactive CD8+ T cell responses through independent, recombination events and the recombinants were selected because they afforded a replication advantage under dual T cell selection pressure.

### Classical T cell escape of variable epitope occurred following recombination

As infection progressed, initial, partial escape from the T cell responses targeting two Nef epitopes through recombination was followed by classic escape, i.e. the emergence of *de novo* mutations (not present in either T/F virus and not derived through recombination) within the targeted epitopes, conferring complete escape. The R71T change at position 4 in the Nef68-76 epitope, which was first detected at day 134 and was present in 100% of SGA sequences at day 441, abrogated all detectable T cell recognition (Figure 4A). In the Nef 19–27 epitope, additional intra-epitope R19K and R21K changes also independently ablated T cell recognition (Figure 4C).

## Discussion

Two major determinants of virus escape patterns are immunodominance and epitope entropy (population level sequence variability) [10,11]. In CH078, the early immunodominant Rev-specific T cell response which targeted a high entropy epitope escaped within weeks (Figure 3). Escape in this epitope occurred classically through the accumulation of non-synonymous mutations within the epitope. Conversely, the acute CD8+ T cell response targeting the Gag TL9 epitope which is very low entropy did not escape over the study window, despite subsequently gaining immunodominance. Slow escape in the TL9 epitope has been described elsewhere [33].

By contrast, the Nef68-76 CD8+ T cell response, which was subdominant and targeted a low entropy epitope, both parameters associated with slow virus escape [10], escaped rapidly, within weeks of infection. Here, recombination provided an accelerated escape in acute HIV-1 infection. The efficiency of recombination as a rapid mode of escape from T cell immune pressure in acute HIV-1 infection was highlighted by selection of recombinants that simultaneously excised two Nef epitopes located 50aa apart. Five different recombination patterns were selected within the same short period, strongly implying that direct selective forces were acting on this region as opposed to selection occurring elsewhere in the genome and recombinant strains becoming dominant through linkage between two epitopes with escape mutations.

These observations of rapid recombination and CD8+ T cell escape in acute infection are novel. They add to, but are distinct from, two previous reports that describe superinfection in the chronic stage of HIV-1 infection. Those studies described the selection of the superinfecting strain because it contained mutations that conferred escape from circulating T cell responses [29,34]. In one of these studies, recombination was detected in a patient two months after the superinfection event, and was associated with T cell pressure [29]. Here, our more detailed sampling clearly shows that primary HIV-1 specific T cell responses can select recombination between distinct T/F viruses even more rapidly, within weeks of infection when pVL is stabilizing prior to the establishment of setpoint. These observations provide empirical evidence of a mechanism beyond stochastic events to explain the emergence of recombination reported in acute infection [12,14,15,17]. A striking feature of the results presented here are the five independent recombinations observed in a very short time period and the fact that two separate T cell responses, neither immunodominant, combined to select the recombinations.

Very interestingly, as infection progressed the early recombination mediated escape in the Nef epitopes was followed by the emergence of non-synonymous mutations within the targeted epitopes that themselves more strongly ablated CD8 T cell recognition. This is consistent with recombination offering not only an alternate route of escape but in some cases more rapid escape.

Our observations are also consistent with others [35,36], that show T cell escape is a dynamic process. In addition to the ongoing emergence of HIV-1 variants through either recombination or mutation, we also observed changing recognition of T cell escape variants over time (Figure 3, Additional file 5: Supplementary Text). This is best explained by the emergence of discrete circulating T cell clonotypes that differentially recognized epitopes as HIV-1 infection progressed [33]. These data underscore the complexity of interpreting T cell selection pressure in chronic HIV-1 infection.

Although it seems likely that the selection of recombinants in some patients may have negative outcomes for the host, this did not occur in this patient, at least in the study window observed. Other host responses, including the subject’s more dominant T cell responses which exhibited consistent oligofunctionality, could have contributed to the virus control observed; particularly as this subject expressed the HLA B*81 allele which is overrepresented in viremic controllers [37]. Other viremic factors, variation in Nef-mediated immune evasion [38,39] or Gag-Protease replicative capacity [40] could have resulted in emergence of less fit and or less pathogenic viruses resulting in the lower setpoint. Given the number of variables in this, a single patient study, we cannot draw firm conclusions regarding the HIV-1 control observed.

## Conclusions

This in depth case study of a patient infected with two T/F HIV-1 viruses has demonstrated that the appearance of recombinants known to occur during acute infection [12,14,15,17] can be driven by the selective action of CD8+ T cell responses.

These observations have clinical implications. Whilst we show that recombination mediated escape can occur within weeks of infection, the generation of escape mutants will be generally slower in regions of sequence conservation. Vaccine-induced T cell responses that dominantly target conserved regions of HIV-1 will therefore also be less subject to rapid escape by recombination suggesting a conserved immunogen design for prophylactic T cell vaccines could also retain benefit in individuals infected with > 1 HIV-1 T/F virus. Functional cure of HIV-1 is an emerging field with a role for HIV-1 specific CD8 T cells to detect and inhibit HIV-1 reservoirs recently highlighted [41]. Our observations suggest that following reactivation of the HIV-1 reservoir, recombination between proviruses, even in individuals who received very early antiretroviral therapy, could mediate very rapid escape from T cell responses.

## Methods

### Study subject

CHAVI patient 078 is a black South African male aged 22 at the time of screening. Transmission occurred via heterosexual sex from a known HIV-1^+^ patient. The subject remained antiretroviral naive over the subsequent study period of 442 days. At screening, approximately 22 days post transmission, samples were collected for viral identification and serology only. Twenty-one days post-screening, the patient was enrolled into the acute infection arm of the Center for HIV/AIDs Vaccine Immunology (CHAVI) 001 study. At enrolment and all subsequent visits, blood was drawn, PBMCs isolated, and these cells used for subsequent T cell studies. Plasma and sera were also isolated for viral sequencing at these visits.

### Amplification of near full-length viral genome by SGA

Viral RNA was extracted from longitudinal plasma samples using the PureLink Viral RNA/DNA Mini Kit (Invitrogen, Carlsbad, CA). cDNA was synthesized using the SuperScript III reverse transcriptase (Invitrogen, Carlsbad, CA) with the primers 07Rev8 5′- CCTARTGGGATGTGTACTTCTGAACTT-3′ (nt5193-5219 in HXB2) for the 5′ half genome, or primer 1.R3.B3R 5′- ACTACTTGAAGCACTCAAGGCAAGCTTTATTG-3′ (nt9611-9642) for the 3′ half genome. Single genome amplification (SGA) was performed to obtain the 5′ half, 3′ half or near full-length HIV-1 genome as described previously [13,42]. For the 5′ half genome amplification, the first round PCR was carried out using the primers 1.U5.B1F 5′-CCTTGAGTGCTTCAAGTAGTGTGTGCCCGTCTGT-3′ (nt538-571) and 07Rev8 5′-CCTARTGGGATGTGTACTTCTGAACTT-3′ (nt5193-5219), and the second round PCR with primers Upper1A 5′-AGTGGCGCCCGAACAGG-3′ (nt634-650) and Rev11 5′-ATCATCACCTGCCATCTGTTTTCCAT-3′ (nt5041-5066). To amplify the 3′ half genome, the first round PCR was performed using the primers 07For7 5′-CAAATTAYAAAAATTCAAAATTTTCGGGTTTATTACAG-3′ (nt 4875–4912) and 2.R3.B6R 5′-TGAAGCACTCAAGGCAAGCTTTATTGAGGC-3′ (nt9636-9607), and the second round PCR with primers VIF1 5′-GGGTTTATTACAGGGACAGCAGAG-3′ (nt4900-4923) and Low2c 5′-TGAGGCTTAAGCAGTGGGTTCC-3′ (nt9591-9612).

### Sequence analysis

The SGA amplicons were directly sequenced by the cycle sequencing and dye terminator methods on an ABI 3730xl genetic analyzer (Applied Biosystems, Foster City, CA). Individual sequences were assembled and edited using Sequencher 4.7 (Gene Codes, Ann Arbor, MI). The sequences were aligned using CLUSTAL W [43] and the manual adjustment for optimal alignment was performed using Seaview. The Neighbor-joining (NJ) tree was constructed using the Kimura 2-parameter model. The highlighter plot was generated using the Highlighter tool at the Los Alamos HIV sequence database (http://www.hiv.lanl.gov/content/sequence/HIGHLIGHT/highlighter_top.html). Genbank accession numbers: 3′ sequences KC149035-149139, 5′ sequences KC148775-149034.

### Recombination analysis

All sequences from different time points were compared to the parental T/F sequences. Recombination breakpoints were identified by RDP3 followed by manually inspection of the Highlighter plot. The aligned sequences were analyzed for recombination signals using BOOTSCAN, GENECONV, MAXCHI, CHIMAERA, SISCAN and 3SEQ in the program RDP3 with a window size of 20 nucleosides [44]. The T/F sequences were used as the parental sequences.

### T cell response analysis

IFN-γ ELISpot mapping, Shannon entropy calculations [45] and time to T cell escape were performed as previously described [10]. For mapping, the entire proteome of both T/F viruses were tested using PBMCs from days 28 and 187, while responses against both NEF proteomes were additionally tested for at days 42 and 105. To investigate whether ‘novel’ T cell responses were induced in response to viral diversification, variant peptides that matched non-synonymous ‘stripes’ that emerged over the course of the study window were tested. No *de novo* T cell responses were detected.

Once all optimal epitopes had been identified, these were tested at 1 μM concentrations as outlined across multiple visits using *ex vivo* IFN-γ ELISpots and flow cytometry. Avidity of responses was tested at some timepoints using peptide dilutions. Peptides are identified by alignment to HXB2 proteins.

### Flow cytometry

Following thawing and O/N resting, PBMCs were stimulated with peptide or control (0.45% DMSO) for 6 hrs in the presence of Costim™ (BD Biosciences) plus Brefeldin A and Monensin. Anti-CD107a fluorochrome-labelled antibody was also added to assay for degranulation. Cells were then washed with PBS and incubated with LIVE/DEAD Aqua viability dye (Invitrogen Life Technologies) followed by fluorochrome labelled antibodies against CD4, CD8, CD19, CD45RO and CD27. Cells were fixed and permeabilised using Cytofix™ prior to staining with fluorochrome-labelled antibodies against CD3, IFN-γ, TNF-α and IL-2. Flow cytometry was performed on BD LSRII and data was analysed using FlowJo (Tree Star Inc.) using the following gating strategy lymphocytes/singlets/alive/CD19-CD3+/CD4-CD8+/exclusion of CD27 + CD45RO- cells. Polyfunctionality was analysed using Pestle and SPICE software from Dr Mario Roederer, NIH. Data shown is background subtracted and a positive responses defined the mean plus two standard deviations of mock-stimulated response measured across all timepoints (n = 7) tested.

### Ethics Statement

CHAVI 078 gave full, written consent to enrol into the acute infection arm of CHAVI 001. Experiments were approved by the Prevention Sciences Review Committee, Division of Acquired Immunodeficiency Syndrome and by the Oxford Tropical Research Ethics Committee.

## Additional files

Additional file 1: Figure S1. Highlighter analysis of sequential half genome sequences from subject CH078. 5′ and 3′ half genomes overlapping by 118bp were amplified by SGA from sequential plasma viral RNA. The Highlighter plots denote the location of nucleotide substitutions compared to the sequence representing the inferred major T/F virus. The days post screening (Fiebig I/II) are indicated at the right of the plot. Nucleotide substitutions and gaps are color-coded. Gene locations are indicated beneath each highlighter plot. For the 5′ half of the genome, the presumed minor T/F virus is noted in the 28 days post-screening data and for the 3′ half of the genome, the minor T/F virus noted in the 0 days post-screening data. Additional file 2: Figure S2. T cell responses were CD8+ restricted and primarily of the CD45RO+CD27+ central memory phenotype. Cumulative data on the expression of CD45RO and CD27 by IFN-γ producing CD8+ T cells from intracellular cytokine flow cytometry for each positive peptide response (n=21) observed over study visits between days 21 and 187 from screening/Fiebig I-II. Data are grouped to show T cell phenotypes in response to stimulation with epitopes found in either the major or minor T/F viruses as well as subsequent escape variants. The percentage of central memory (CD27+CD45RO+), effector memory (CD27-CD45RO+), and terminal effector (CD27-CD45RO-) cells within the IFN-γ+ CD8+ T cell population are shown. Bars represent the mean ± 1 standard deviation for each memory population. Additional file 3: Figure S3. CD8+ T cells express IFN-γ, TNF-α and the degranulation marker CD107a in response to both the major and minor variants of Nef68-76. Intracellular cytokines and CD107a expression by CH078 PBMC were assayed by flow cytometry after 6 hours of stimulation with 2μg/ml peptide representing either the major (FPVRPQVPL) or minor (FPVKPQVPL) epitope variants of Nef68-76. A) Single function analysis. Percentage of CD8 memory T cells expressing CD107a (red), IFN-γ (purple) and TNF-α (green) in response to the major (solid line) or minor (dotted line) epitope variants at days 49, 77 and 134 post-screening (Fiebig I/II). B) The proportions of the specific CD8+ memory T cell response accounted for by cells producing all possible combinations of IFNγ, TNFα, IL-2 and CD107a. Colored arcs indicate expression of each individual marker and the shaded sectors show the proportion of cells expressing each marker combination. Additional file 4: Figure S4. Highlighter recombination plot of partial *nef* gene sequence. The first 342 bp of the *nef* gene sequence containing both Nef T cell epitopes were used to detect recombination events. The major and minor T/F viral sequences are shown in blue and red lines at the top, respectively. The major and minor T/F virus signature nucleotides are indicated as blue and red ticks, respectively. The nucleotides that differ from both T/F viruses are indicated as black ticks. The Nef T cell epitopes are outlined in green box. The days post-screening (Fiebig I/II) are indicated at the right of the plot. Additional file 5: Supplementary Text. Maturation of classic virus escape. As previously described, the emergence of virus variants within T cell epitopes is not static and changes over time [9,10,35]. Given our access to longitudinal samples, we examined how the more common epitope variants that emerged at different stages following infection impacted T cell recognition in the shared epitopes. Several patterns were observed. In the early immunodominant Rev12-20 epitope, the most effective R17K escape remained dominant in the virus population and the less effective K20R variant disappeared by day 77 (Figure 3A). In 2 epitopes (Gag25-34, Nef68-76), maturation of virus escape was observed with different escape variants dominant at earlier and later timepoints with the later escapes more effectively ablating T cell recognition (Figures 3D and 4A). These examples suggest that under ongoing T cell selection, HIV-1 ultimately selected the most effective virus escape. Interestingly, in a fourth epitope (Vif73-81), the early A78D mutation ablated the T cell response much more strongly than the P75T mutation which arose later in infection. This hierarchy of recognition changed with time. At day 266, the P75T mutation ablated T cell recognition far more effectively than the A78D mutation (Figure 3C). The changing recognition by T cells of escape variants over time, suggests the emergence of discrete circulating T cell clonotypes that differentially recognize these epitopes, recently described in chronic HIV-1 infection in [33]. Therefore, not only does HIV-1 evolve in response to T cell responses, but T cell responses themselves also adapt in response to virus escape. These data underscore the complexity of interpreting T cell selection pressure in chronic HIV-1 infection.

## Abbreviations

aa: Amino acid
HIV-1: Human immunodeficiency virus-1
HLA: Human leukocyte antigen
PBMC: Peripheral blood mononuclear cell
O/N: Overnight
SGA: Single genome amplification
SFU: Spot forming unit
T/F: Transmitted/founder
pVL: Virus load
