# Supplementary figures and images for: Recombination-mediated escape from primary CD8+ T cells in acute HIV-1 infection
Source: Retrovirology. 2014 Sep 12;11:69. doi: 10.1186/s12977-014-0069-9 (PMC4180588; doi:10.1186/s12977-014-0069-9)

## Slide 1
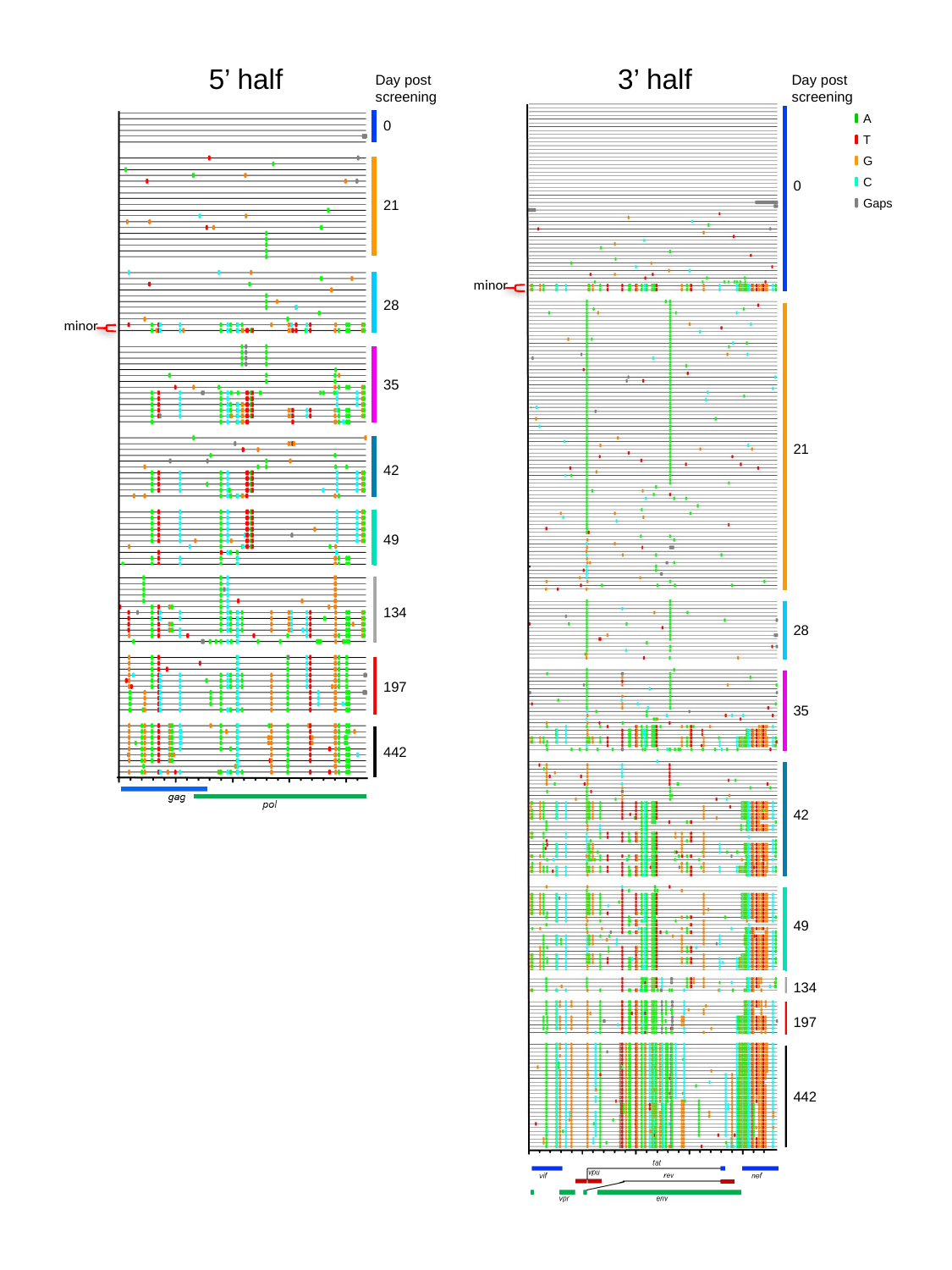

5’ half
3’ half
Day post
screening
Day post
screening
A
0
T
G
C
0
Gaps
21
minor
28
minor
35
21
42
49
134
28
197
35
442
42
49
134
197
442

Supplement: Additional file 1: Figure S1. — Highlighter analysis of sequential half genome sequences from subject CH078. 5′ and 3′ half genomes overlapping by 118bp were amplified by SGA from sequential plasma viral RNA. The Highlighter plots denote the location of nucleotide substitutions compared to the sequence representing the inferred major T/F virus. The days post screening (Fiebig I/II) are indicated at the right of the plot. Nucleotide substitutions and gaps are color-coded. Gene locations are indicated beneath each highlighter plot. For the 5′ half of the genome, the presumed minor T/F virus is noted in the 28 days post-screening data and for the 3′ half of the genome, the minor T/F virus noted in the 0 days post-screening data. [file 12977_2014_69_MOESM1_ESM.pptx]

Percentage of CD8+ IFN- $\gamma$ + T cells

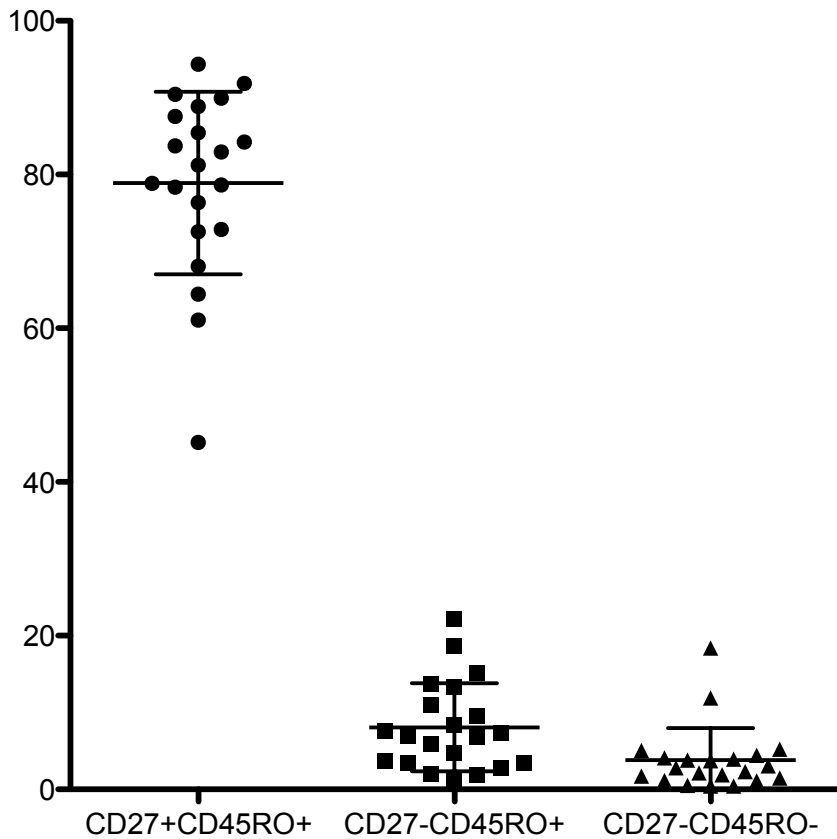

Supplement: Additional file 2: Figure S2. — T cell responses were CD8+ restricted and primarily of the CD45RO+CD27+ central memory phenotype. Cumulative data on the expression of CD45RO and CD27 by IFN-γ producing CD8+ T cells from intracellular cytokine flow cytometry for each positive peptide response (n=21) observed over study visits between days 21 and 187 from screening/Fiebig I-II. Data are grouped to show T cell phenotypes in response to stimulation with epitopes found in either the major or minor T/F viruses as well as subsequent escape variants. The percentage of central memory (CD27+CD45RO+), effector memory (CD27-CD45RO+), and terminal effector (CD27-CD45RO-) cells within the IFN-γ+ CD8+ T cell population are shown. Bars represent the mean ± 1 standard deviation for each memory population. [file 12977_2014_69_MOESM2_ESM.pdf]

# A

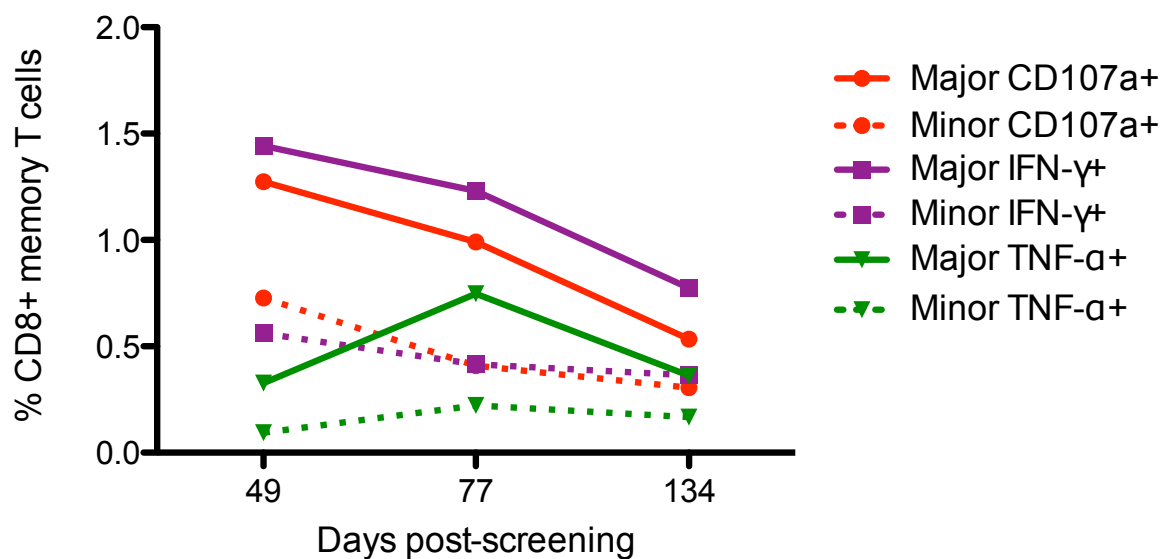

# B

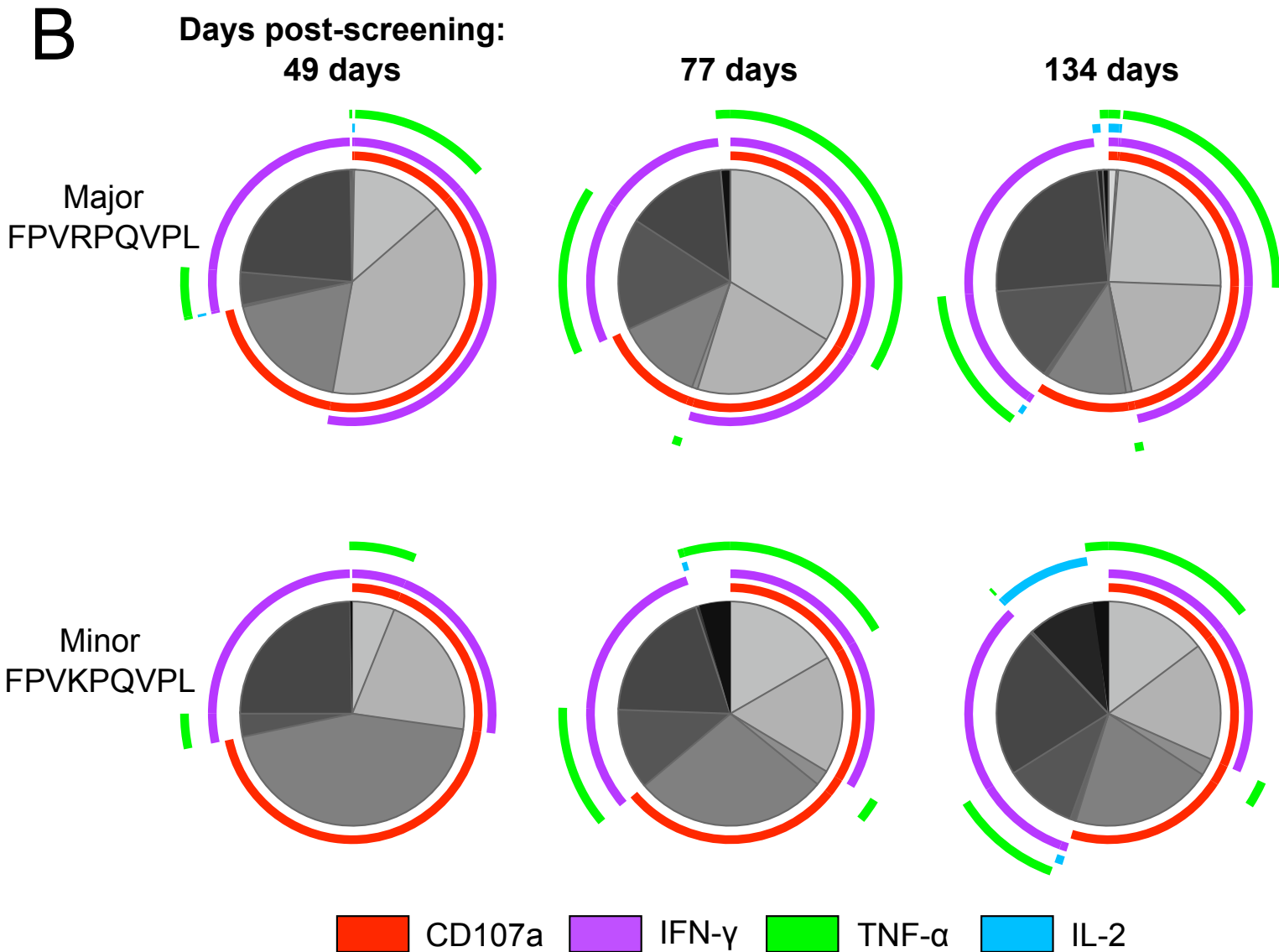

Supplement: Additional file 3: Figure S3. — CD8+ T cells express IFN-γ, TNF-α and the degranulation marker CD107a in response to both the major and minor variants of Nef68-76. Intracellular cytokines and CD107a expression by CH078 PBMC were assayed by flow cytometry after 6 hours of stimulation with 2μg/ml peptide representing either the major (FPVRPQVPL) or minor (FPVKPQVPL) epitope variants of Nef68-76. A) Single function analysis. Percentage of CD8 memory T cells expressing CD107a (red), IFN-γ (purple) and TNF-α (green) in response to the major (solid line) or minor (dotted line) epitope variants at days 49, 77 and 134 post-screening (Fiebig I/II). B) The proportions of the specific CD8+ memory T cell response accounted for by cells producing all possible combinations of IFNγ, TNFα, IL-2 and CD107a. Colored arcs indicate expression of each individual marker and the shaded sectors show the proportion of cells expressing each marker combination. [file 12977_2014_69_MOESM3_ESM.pdf]

## Slide 1
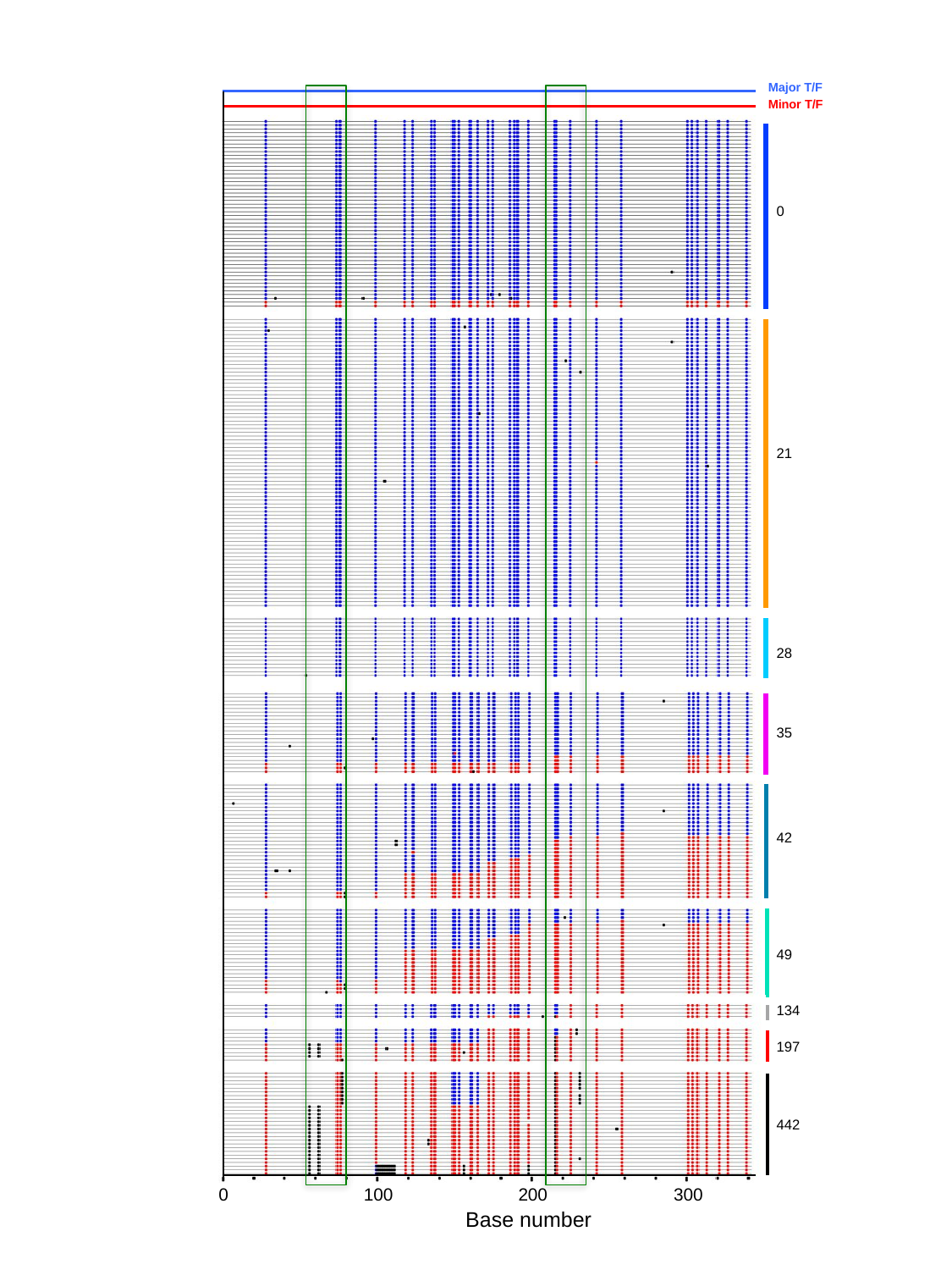

Major T/F
Minor T/F
0
21
28
35
42
49
134
197
442
0
100
200
300
Base number

Supplement: Additional file 4: Figure S4. — Highlighter recombination plot of partial nef gene sequence. The first 342 bp of the nef gene sequence containing both Nef T cell epitopes were used to detect recombination events. The major and minor T/F viral sequences are shown in blue and red lines at the top, respectively. The major and minor T/F virus signature nucleotides are indicated as blue and red ticks, respectively. The nucleotides that differ from both T/F viruses are indicated as black ticks. The Nef T cell epitopes are outlined in green box. The days post-screening (Fiebig I/II) are indicated at the right of the plot. [file 12977_2014_69_MOESM4_ESM.pptx]
