# Supplementary material for: Recombination-mediated escape from primary CD8+ T cells in acute HIV-1 infection
Source: Retrovirology. 2014 Sep 12;11:69. doi: 10.1186/s12977-014-0069-9 (PMC4180588; doi:10.1186/s12977-014-0069-9)
Supplement: Additional file 5: Supplementary Text. — Maturation of classic virus escape. As previously described, the emergence of virus variants within T cell epitopes is not static and changes over time [9,10,35]. Given our access to longitudinal samples, we examined how the more common epitope variants that emerged at different stages following infection impacted T cell recognition in the shared epitopes. Several patterns were observed. In the early immunodominant Rev12-20 epitope, the most effective R17K escape remained dominant in the virus population and the less effective K20R variant disappeared by day 77 (Figure 3A). In 2 epitopes (Gag25-34, Nef68-76), maturation of virus escape was observed with different escape variants dominant at earlier and later timepoints with the later escapes more effectively ablating T cell recognition (Figures 3D and 4A). These examples suggest that under ongoing T cell selection, HIV-1 ultimately selected the most effective virus escape. Interestingly, in a fourth epitope (Vif73-81), the early A78D mutation ablated the T cell response much more strongly than the P75T mutation which arose later in infection. This hierarchy of recognition changed with time. At day 266, the P75T mutation ablated T cell recognition far more effectively than the A78D mutation (Figure 3C). The changing recognition by T cells of escape variants over time, suggests the emergence of discrete circulating T cell clonotypes that differentially recognize these epitopes, recently described in chronic HIV-1 infection in [33]. Therefore, not only does HIV-1 evolve in response to T cell responses, but T cell responses themselves also adapt in response to virus escape. These data underscore the complexity of interpreting T cell selection pressure in chronic HIV-1 infection. [file 12977_2014_69_MOESM5_ESM.docx]

Supplementary Text: Maturation of classic virus escape

As previously described, the emergence of virus variants within T cell epitopes is not static and changes over time [[9](#_ENREF_9), [10](#_ENREF_10), [35](#_ENREF_35)]. Given our access to longitudinal samples, we examined how the more common epitope variants that emerged at different stages following infection impacted T cell recognition in the shared epitopes. Several patterns were observed. In the early immunodominant Rev12-20 epitope, the most effective R17K escape remained dominant and the less effective K20R variant disappeared by day 77 (Fig 3A). In 2 epitopes (Gag25-34, Nef68-76), maturation of virus escape was observed with different escape variants dominant at earlier and later timepoints with the later escapes more effectively ablating T cell recognition (Fig 3D 4A). These examples suggest that under ongoing T cell selection, HIV-1 ultimately selected the most effective virus escape. Interestingly, in a fourth epitope (Vif73-81), the A78D variant was first detected at day 35 then declined over time while the later P75T variant was first detected day 42 but was found in all SGA sequences at day 442. During acute infection, the A78D mutation ablated the T cell response much more strongly than the P75T mutation. This hierarchy of recognition changed with time. At day 266, the P75T mutation ablated T cell recognition far more effectively than the A78D mutation (66% vs. 10% respectively) (Fig 3C). The changing recognition by T cells of escape variants over time, suggests the emergence of discrete circulating T cell clonotypes that differentially recognise these epitopes, recently described in chronic HIV-1 infection in [[33](#_ENREF_33)]. Therefore, not only does HIV-1 evolve in response to T cell responses, but T cell responses themselves also adapt in response to virus escape. These data underscore the complexity of interpreting T cell selection pressure in chronic HIV-1 infection.
